# Effect of Chlorine Dioxide Treatment on Human Pathogens on Iceberg Lettuce

**DOI:** 10.3390/foods10030574

**Published:** 2021-03-10

**Authors:** Karin Hassenberg, Ulrike Praeger, Werner B. Herppich

**Affiliations:** Department of Horticultural Engineering, Leibniz Institute for Agricultural Engineering and Bioeconomy (ATB), Max-Eyth-Allee 100, 14469 Potsdam, Germany; upraeger@atb-potsdam.de (U.P.); wherppich@atb-potsdam.de (W.B.H.)

**Keywords:** chemical prevention, cross-contamination, *Escherichia coli*, *Salmonella enterica*, *Listeria monocytogenes*

## Abstract

In the vegetable processing industry, the application of chlorine dioxide (ClO_2_) as a disinfectant solved in washing water to eliminate undesirable microorganisms harmful to consumers’ health and the shelf life of produce has been discussed for years. The disinfection efficacy depends on various factors, e.g., the location of microorganisms and the organic load of the washing water. The present study analyzed the sanitation efficacy of various concentrations of water-solved ClO_2_ (cClO_2_: 20 and 30 mg L^−1^) on *Escherichia coli* (1.1 × 10^4^ cfu mL^−1^), *Salmonella enterica* (2.0 × 10^4^ cfu mL^−1^) and *Listeria monocytogenes* (1.7 × 10^5^ cfu mL^−1^) loads, located on the leaf surface of iceberg lettuce assigned for fresh-cut salads. In addition, it examined the potential of ClO_2_ to prevent the cross-contamination of these microbes in lettuce washing water containing a chemical oxygen demand (COD) content of 350 mg L^−1^ after practice-relevant washing times of 1 and 2 min. On iceberg leaves, washing with 30 mg L^−1^ ClO_2_ pronouncedly (1 log) reduced loads of *E. coli* and *S. enterica*, while it only insignificantly (<0.5 × log) diminished the loads of *L. monocytogenes*, irrespective of the ClO_2_ concentration used. Although the sanitation efficacy of ClO_2_ washing was only limited, the addition of ClO_2_ to the washing water avoided cross-contamination even at high organic loads. Thus, the application of ClO_2_ to the lettuce washing water can improve product quality and consumer safety.

## 1. Introduction

During recent years, the consumption of minimally processed fresh-cut salads has rapidly increased, with growth rates of 10 to 20% per year [1,2]. With the enhanced popularity of “ready-to-eat” produce, the number of reports on outbreaks of food-borne diseases has multiplied. Many of them are caused by human pathogens on fresh vegetables and salads [3]. The causes can be traced back to cultivation conditions and processing. During cultivation, vegetables can be contaminated by the excrement of wild animals or by wastewater irrigation. During processing, produce can be contaminated by deficient personnel and industrial hygiene and the cross-contamination of produce [4,5].

Production processes of ready-to-eat fresh-cut salads are very simply designed. The vegetables are sorted, cut, washed, dried and packed in plastic bags. However, it is known that washing with tap water results in a 10- to 100-fold reduction of microorganisms on the produce surface [6]. On romaine or iceberg lettuce, washing with plain water without sanitizing treatments reduces the microorganisms by ≤1 × log cfu g^−1^ [7,8].

To guarantee a high quality of produce until the end of the minimum shelf life, the Committee of Food Microbiology and Sanitation of the German Association for Sanitation and Microbiology released guidance and warning limits for the relevant microbial contaminations of packed fresh-cut salads [9]. Producers of ready-to-eat salads must warrant these limits until the end of the minimum guaranteed shelf life, but they cannot any longer control the produce or its storage conditions once it has left the production site. Interruptions in the cooling chains of these highly perishable products easily result in the substantial growth of spoilage and human pathogens. Visual detection of human pathogens by the consumer is not possible during purchasing, and their decision is normally guided by sensory criteria such as the freshness and crisp appearance of the salad [10,11].

Therefore, in recent years, several chemical and physical treatments have been investigated and applied with the aim to reduce the initial microbiological load of human pathogens on produce. For instance, the application of chlorine is a usual practice worldwide, but the reducing effect on microorganisms is not as effective as hoped [12] and causes the formation of hazardous byproducts, such as carcinogenic trihalomethane (THM) in food [13]. As a consequence, the application of chlorine is forbidden by German law [14]. Ozone has been used as an antimicrobial agent since the 19th century in drinking water purification and for a long time for vegetable and fruit disinfection [15]. It can be applied in an aqueous or gaseous form and is effective over a much wider spectrum of microorganisms than chlorine and other disinfectants [16]. However, exposure to high ozone concentrations can cause some detrimental health effects. Therefore, many countries specified thresholds for continuous exposure in the workplace environment, and effective and reliable ozone monitoring and alarm systems must be installed for workers’ safety [17,18]. Ozone application may also cause undesirable effects on treated produce such as oxidative stress or physiological injuries such as mass loss, browning or discoloration [19,20]. The use of various different organic acids was, furthermore, tested. Acetic acid is a strong oxidant, but its application should be harmless, based on the fact that acetic acid is a universal metabolic intermediary and occurs in plants and animals. Several studies, however, observed undesirable effects on produce quality; so, acetic acid fumigation is not a suitable technique for fresh produce treatment [21,22,23]. Other studies indicated that peroxyacetic acid can negatively affect the quality of produce [24]. Moreover, the application of high doses of UV-C radiation may also negatively affect produce quality, e.g., resulting in discoloration, browning or a decrease in vitamin C content [25].

The development of alternative sanitation techniques is, therefore, of great interest. In this context, chlorine dioxide (ClO_2_) is of increasing interest for fresh produce. It is a strong bactericide and virucide with a high oxidative capacity [26,27] and produces much fewer harmful byproducts such as trihalomethanes or chloramines than, e.g., chlorine [6,28]. The antibacterial effect of ClO_2_ is almost independent of the pH value (between 3 and 8) in contrast to chlorine [29].

Only a few studies indicated some negative impacts of aqueous ClO_2_ treatment on the visual and/or sensory quality and shelf life of produce such as lettuce [8], while many others proved the better maintenance of the sensory quality of lettuce and some berry fruits by this treatment, e.g., compared to tap water washing [30,31,32,33].

Chlorine dioxide can be applied dissolved in water or as gas. Gaseous application is more effective against microbial loads because of the higher penetrability of the gas [34], which, however, is rather explosive. Therefore, the aqueous application is certainly preferable. Hence, the present study compares the efficacy of washing with chlorine dioxide solution relative to that of conventional tap water on human pathogens as an alternative sanitizer on iceberg lettuce. For this, artificially inoculated lettuce leaves were washed with both methods, and their respective effects on relevant human pathogens were evaluated. In addition, the ability of water-solved ClO_2_ to prevent cross-contamination under practical conditions was analyzed in lettuce washing water prepared with a chemical oxygen demand (COD) of 350 mg L^−1^.

## 2. Materials and Methods

For the study, cryo bead-stored (Carl Roth GmbH, Karlsruhe, Germany) samples (at −80 °C) of *Escherichia coli* (DSMZ 19206), *Listeria monocytogenes* (DSMZ 20600) and *Salmonella enterica* (DSMZ 17058) were incubated in nutrient broth (5 mL; NB; Carl Roth GmbH, Karlsruhe, Deutschland; *E. coli* and *S. enterica*) and brain heart infusion broth (BHIB; Carl Roth GmbH, Karlsruhe, Germany; *L. monocytogenes*) at 37 °C for 24 h, and the optical density of bacterial suspensions was determined using a BioPhotometer plus (Eppendorf, Hamburg, Germany) at 600 nm. According to the optical density, the main culture was inoculated in NB or BHIB, respectively, and shaken at 170 rpm and 38 °C for 18 h, resulting in bacterial suspensions of approx. 10^9^ cfu mL^−1^, cell counted (Multisizer™ 3 Coulter Counter^®^, Beckman Coulter, Krefeld, Germany) and further diluted to yield initial bacterial counts of approx. 10^7^ cfu mL^−1^.

For the experiment, fresh PVC bags packed with iceberg lettuce heads were obtained from a local retailer and brought to the laboratory. There, the outer leaves were removed and discarded, and 10 g of the inner leaves were used for experiments. For this, the leaves were inoculated with 1 mL of bacteria suspension by spraying and then stored for 2 h in a clean bench for the establishment of microorganisms on the leaf surfaces with an average concentration of *E. coli* of 1.1 × 10^4^ cfu mL^−1^, *S. enterica* of 2.0 × 10^4^ cfu mL^−1^ and *L. monocytogenes* of 1.7 × 10^5^ cfu mL^−1^.

The Dr. Küke two-component product (Dr. Küke GmbH, Hannover, Germany) was used to produce chlorine dioxide. Following the manufacturer’s instructions, 250 mL of Component 1 (“DK-DOX aktiv”) was mixed with 3.85 g of Component 2 (DK-DOX) for activation. After a reaction time of 24 h (30 °C), the fully activated product was ready for use. ClO_2_ concentrations were determined photometrically (spectral photometer: DR 2800; cuvette test: LCK 310, both Hach Lange, Berlin, Germany), and the stock solution was diluted with tap water to adjust the required concentration.

For cross-contamination experiments, synthetic salad washing water was prepared by halving an iceberg salad head, discarding the core, cutting the remaining head into small pieces, puréeing it with a handheld blender and finally squeezing it through a tea strainer. Filtration through a PE filter (pore size: 330 µm) removed the remaining solid matter from the juice. The COD of the filtrate was measured photometrically (spectral photometer: CADAS 200, Dr. Lange, Berlin, Germany; thermostat LT 200, cuvette test LCK 014, both Hach Lange, Berlin, Germany) following the manufacturer’s instructions, and the requested COD was adjusted by adding tap water.

Inactivation tests were always performed in beakers. ClO_2_ solutions (200 mL; cClO_2_ = 0, 20 and 30 mg L^−1^) were filled in the cups, and two inoculated lettuce leaves were added. The cup’s contents were carefully blended, and after 1 or 2 min, respectively, equivalent amounts of sodium thiosulfate pentahydrate (AppliChem, Darmstadt, Germany) were added to stop inactivation. Subsequently, all samples were washed with tap water and carefully blotted dry with paper towels. The effect of the treatments was evaluated by counting bacteria with the spread plate method, for which serial dilutions were created. Therefore, lettuce samples (20 g) were mixed with 220 mL of Ringer’s solution (Merck, Darmstadt, Germany) in a stomacher bag and homogenized for 2 min in a stomacher. Afterwards, samples (100 µL) were plated on nutrient agar (Carl Roth GmbH, Karlsruhe, Germany) and incubated at 37 °C for 24 h.

To analyze the effect of preventing cross-contamination by chlorine dioxide treatment, the following procedure was carried out. For analyses, water samples (1 L) were prepared from lettuce sap and bacteria and final average concentrations of 350 mg L^−1^ (COD), 2.0 10^4^ cfu mL^−1^ (*E. coli*), 3.3 10^5^ cfu mL^−1^ (*S. enterica*) and 8.7 10^4^ cfu mL^−1^ (*L. monocytogenes*). Then, six lettuce leaves (10 g) and, immediately afterwards, ClO_2_ solutions (final concentration: 20 or 30 mg L^−1^), were added. After 1 or 2 min, the lettuce leaves were removed, washed with tap water and carefully blotted dry with paper towels. Subsequently, the microbiological counts of the lettuce samples were analyzed by counting bacteria with the spread plate method as described above. For each experiment, untreated lettuce leaves were analyzed as controls, as well as by counting bacteria with the spread plate method as described above. All experiments were repeated thrice, and all samples were analyzed twice.

Data were statistically analyzed with WinSTAT (R. Fitch Software, Bad Krozingen, Germany). Treatment means were statistically compared using Duncan’s multiple range test (*p* < 0.05).

## 3. Results

### 3.1. Effect of ClO_2_ Treatment on E. coli, S. enterica and L. monocytogenes on Iceberg Lettuce

Compared to controls, all treatments significantly reduced *E. coli* loads, with higher ClO_2_ concentrations tending to improve reduction (Table 1). In this context, a ClO_2_ concentration of 30 mg L^−^^1^ at a 1 min contact time most effectively inactivated *E. coli* (reduction: 88%). Enhancement of the contact time to 2 min did not further improve germ reduction.

Given are means (*n* = 3) ± standard deviation. Different letters indicate the significant difference between means (*p* < 0.05).

All ClO_2_ treatments significantly inactivated *S. enterica*, irrespective of ClO_2_ concentration and treatment time. Nevertheless, chlorine dioxide treatment at a high concentration (30 mg L^−^^1^) and a long treatment time (2 min) yielded the best (90%) reduction of *S. enterica*, although the results of the various treatments were not significantly different.

*L. monocytogenes* proved the most resistant microorganism to ClO_2_ treatment, which only insignificantly reduced *L. monocytogenes* loads by 58 to 72% compared to the controls.

### 3.2. Effect of ClO_2_ Treatment to Prevent Cross-Contamination

Before the experiment, iceberg lettuce leaves were analyzed to obtain the starting concentrations of the respective bacteria on the surfaces (Table 2). After 1 or 2 min of washing in ClO_2_ water, which was contaminated with *E. coli*, *S. enterica* or *L. monocytogenes* at known concentrations, the lettuce leaves were analyzed again. The final concentrations of microorganisms were still in the same range found on the unwashed controls. Adding chlorine dioxide to the contaminated washing water successfully prevented any cross-contamination. In fact, it marginally (and insignificantly) reduced the loads of all microorganisms on the lettuce leaf surface compared to the starting concentrations, except for *S. enterica* at a cClO_2_ of 20 mg L^–1^ and a treatment duration of 2 min (Table 2).

## 4. Discussion

The sanitizing effect of ClO_2_ for human pathogenic bacteria in water has been described in several studies [29,35,36,37,38]. It was shown that sanitation success closely depends on multiple factors, including ClO_2_ concentrations, temperature and duration of treatments or the concentration of organic matter in the water [39,40,41].

Particularly relevant for the sanitation of lettuce for fresh-cut salads is the direct effect of ClO_2_ solved in the washing water on the microorganisms adherent to the leaf surfaces. Some protective sites (biofilms, injuries, etc.) or water-suspended solids and aggregates, and other effects of leaf surfaces may pronouncedly reduce the potential antimicrobial effect of chlorine dioxide compared to bacteria separately floating in the washing water [41,42,43,44]. In addition, the processing status of the produce is relevant for the efficacy of the ClO_2_ treatment. On minimally processed produce such as fresh-cut lettuce or apples, ClO_2_ is less effective in inactivating *E. coli* and *L. monocytogenes* than on intact leaves or fruit [45]. This is mostly due to the increased surface contact area and the wounded tissue. The log reductions of *L. monocytogenes* by aqueous ClO_2_ treatment were significantly higher on uninjured than on surface-injured green pepper fruit [34]. Even on intact produce, however, cracks and other surface injuries may partially protect bacteria against decontamination [40]. Furthermore, due to the physicochemical properties of cuticle and waxy layers, pockets in the leaf epidermis are hydrophobic and, thus, may serve as protective pockets for microbes. Here, the aqueous chlorine dioxide cannot penetrate, and the bacteria remain unaffected [46].

Therefore, the above clearly stresses the great practical importance of the effects of ClO_2_ treatment on relevant microorganisms located on the product surface for the sanitation of fresh-cut produce. In the present study, the experimentally chosen ClO_2_ concentrations and treatment durations only yielded slight (0.5 × log) and insignificant maximum inactivation efficacies for *L. monocytogenes*. Although the maximum reduction of *E. coli* and *S. enterica* was more successful (approx. 1 × log), the application of water-solved ClO_2_ reduction of relevant microorganisms on the product was indeed much lower than that of isolated microorganisms solved in water. Even in washing water with a high COD load (350 mg L^−1^), reductions of microbial loads of up to 5 log were reported for ClO_2_ treatments [39]. The germ-reducing effects of ClO_2_ washing are severely limited on the produce surface, and, thus, the economic benefit must be critically challenged. Obviously, the use of chlorine dioxide seems to be only economically justified if there are additional benefits. Therefore, the present study evaluated the potential ClO_2_ application to prevent cross-contamination during washing, which is an important issue in the practice of industrial food washing. During processing, especially in recirculated water systems, the probability of produce cross-contamination increases with cycle durations. In recirculated water systems, organic matter accumulates more frequently and to a larger amount than when potable or diluted recirculated water is applied [4,38].

Attempts to assess the efficacy of ClO_2_ in preventing cross-contamination are only meaningful under at least semipractical conditions of industrial lettuce washing processes. Thus, presented experiments were performed with synthetic washing water with a realistic COD of 350 mg L^−1^ [40] and relevant ClO_2_ concentrations and treatment durations [39]. The presented results clearly demonstrated the capability of ClO_2_ to diminish the number of microorganisms (by 0.5–1 × log) on the product surface by reducing the microbial load of the washing water. ClO_2_ treatments, thus, obviously contribute to the safety of fresh-cut salads.

Similarly, the immersion of intact tomatoes in ClO_2_-containing (5 ppm) water prevented cross-contamination by *S. enterica* and *Erwinia carotovora* [5]. In addition, 10 s of spray washing with ClO_2_ solution (5 ppm) pronouncedly (4.7 log) reduced the transfer of *Salmonella* from contaminated brushes to the fruit surfaces [47]. The application of ClO_2_ (3 or 5 mg) to highly turbid tomato processing water (NTU turbidity: 0–40) also largely (7 × log) reduced the loads of *S. enterica* in a broad temperature range (25–40 °C) [47]. At a pilot-scale level, the addition of ClO_2_ (5 and 3 mg L^−1^) to “Lollo Rossa” lettuce washing water successfully minimized *E. coli* cross-contamination [48]; the organic contamination of the washing water, however, typically consumes ClO_2_ to effective concentrations of ≥ 2.5 mg L^−1^. In contrast, the ClO_2_ (3 mg L^−1^) washing of fresh-cut red chard prevents the processing of water cross-contamination from inoculated leaves only by *E. coli* but not *S. enterica* [49]. Additionally, the application of ClO_2_ effectively removes pesticide residues on lettuce leaves and in the washing water [50].

In summary, the efficacy of ClO_2_ to reduce human pathogens on the surface of lettuce leaves is limited; cross-contamination via processing water, however, can be successfully prevented. The overall positive effects recommend the application of ClO_2_ in fresh and fresh-cut lettuce washing systems. Suspended solids and organic matter in the water, however, enhance the protection of microorganisms against sanitation agents. Thus, the removal of dirt particles and pollution by coagulation, sedimentation and filtration is urgently necessary [51], and the application of ClO_2_ for the disinfection of lettuce washing water can be particularly advised for a second washing cycle after the thorough elimination of the pollution.

## Figures and Tables

**Table 1:** Effects of ClO_2_ treatments, varying in concentrations (cClO_2_) and duration, on the inactivation of *E. coli*, *S. enterica* and *L. monocytogenes* on iceberg lettuce leaves in washing water.

	Duration (min)		
	0 (Control)	1	2
**cClO_2_ (mg L^−1^)**	***E. coli* (cfu mL^−1^)**		
0 (control)	^a^ 1.1 × 10^4^ ± 5.1 × 10^3^		
20		^b^ 1.6 × 10^3^ ± 9.5 × 10^2^	^b^ 2.8 × 10^3^ ± 1.7 × 10^3^
30		^b^ 1.3 × 10^3^ ± 1.1 × 10^3^	^b^ 1.5 × 10^3^ ± 1.0 × 10^3^
	***S. enterica* (cfu mL^−1^)**		
0 (control)	^a^ 2.0 × 10^4^ ± 6.5 × 10^3^		
20		^b^ 2.2 × 10^3^ ± 1.1 × 10^3^	^b^ 3.4 × 10^3^ ± 2.4 × 10^3^
30		^b^ 2.3 × 10^3^ ± 4.0 × 10^2^	^b^ 2.0 × 10^3^ ± 9.7 × 10^2^
	***L. monocytogenes* (cfu mL^−1^)**		
0 (control)	^a^ 1.7 × 10^5^ ± 6.5 × 10^3^		
20		^a^ 5.5 × 10^4^ ± 2.9 × 10^4^	^a^ 4.8 × 10^4^ ± 2.4 × 10^4^
30		^a^ 5.0 × 10^4^ ± 2.0 × 10^4^	^a^ 7.2 × 10^4^ ± 5.0 × 10^4^

Given are means (*n* = 3) ± standard deviation. Different letters indicate the significant difference between means (*p* < 0.05).

**Table 2:** Efficacy of adding various concentrations of ClO_2_ to contaminated (*E. coli*, *S. enterica, L. monocytogenes*) washing water (COD 350 mg L^−1^) to prevent the cross-contamination of iceberg lettuce leaves.

Species	Initial Surface Loads (cfu g^−1^)		Loads in Wash Water (cfu mL^−1^)		Surface Loads after Treatment (cfu g^−1^) cClO_2_ (mg L^−1^; Duration (min))	
			20; 1	20; 2	30; 1	30; 2
*E. coli*	^a^ 3.5 × 10^1^ ± 7.0 × 10^0^	2.0 × 10^5^ ± 8.0 × 10^4^	^a^ 1.9 × 10^1^ ± 8.0 × 10^0^	^a^ 1.1 × 10^1^ ± 2.0 × 10^0^	^a^ 1.8 × 10^1^ ± 5.0 × 10^0^	^a^ 2.3 × 10^1^ ± 1.2 × 10^1^
*S. enterica*	^a^ 2.4 × 10^2^ ± 2.9 × 10^2^	3.3 × 10^5^ ± 4.3 × 10^5^	^a^ 1.2 × 10^2^ ± 1.0 × 10^2^	^a^ 7.3 × 10^2^ ± 8.4 × 10^2^	^a^ 5.0 × 10^1^ ± 1.8 × 10^1^	^a^ 4.4 × 10^1^ ± 3.0 × 10^1^
*L. monocytogenes*	^a^ 2.3 × 10^3^ ± 3.2 × 10^3^	8.7 × 10^4^ ± 6.4 × 10^4^	^a^ 9.3 × 10^2^ ± 1.4 × 10^3^	^a^ 3.8 × 10^2^ ± 1.9 × 10^2^	^a^ 4.7 × 10^2^ ± 4.6 × 10^2^	^a^ 2.4 × 10^2^ ± 2.8 × 10^2^

Given are means (*n* = 3) ± standard deviation. Different letters indicate the significant difference between means (*p* < 0.05).

## Data Availability

Data is contained within the article.
